# Decoding the dual roles of monocytes in tumor immunity: from immunosurveillance to immune evasion

**DOI:** 10.3389/fimmu.2026.1805868

**Published:** 2026-04-15

**Authors:** Yi Chang, Yanni Lei, Liang Wang, Liangshuai Liu, Rui Yu

**Affiliations:** 1 Department of Traditional Chinese Medicine, Anshan Central Hospital, Anshan, Liaoning, China; 2 Department of Nursing, Aerospace Central Hospital, Beijing, China; 3 Department of Health and Wellness Medicine, Chinese People's Liberation Army (PLA) General Hospital, Beijing, China; 4 Department of Tuina, Anshan Hospital of Traditional Chinese Medicine, Anshan, Liaoning, China; 5 Department of Cardiology, Liaoning University of Traditional Chinese Medicine, Shenyang, Liaoning, China

**Keywords:** cancer immunotherapy, immune checkpoint inhibitors, immunosuppression, macrophage polarization, monocytes

## Abstract

Monocytes are innate immune cells of the mononuclear phagocyte system, extensively involved in immune and inflammatory responses, and play a critical regulatory role in tumor development and progression. Different monocyte subsets can exert either pro-tumor or anti-tumor functions by modulating immune responses. Through the secretion of cytokines and chemokines, monocytes regulate immune activity, while tumor cells utilize these signaling pathways to influence monocyte polarization, inducing their transformation into immunosuppressive phenotypes. The origin, migration, polarization, and transformation of monocytes within the tumor microenvironment represent key research areas in current cancer immunotherapy. Precise regulation of monocyte function holds promise for developing novel strategies in tumor immunotherapy. Current approaches, such as monocyte-mediated vaccines and combination therapies with immune checkpoint inhibitors, have emerged as major research focuses in cancer immunotherapy. This review summarizes the mechanisms by which monocytes regulate antitumor immune responses and discusses recent advances in their therapeutic applications.

## 1 Introduction

Monocytes originate from hematopoietic stem cells in the bone marrow and subsequently enter the peripheral circulation, where they serve as central regulators of both innate and adaptive immunity. Monocytes comprise heterogeneous subsets with distinct phenotypic markers and developmental trajectories, each mediating specialized immunological functions (1, 2). Through differentiation into effector cell types such as macrophages and dendritic cells, monocytes contribute to both immunostimulatory and immunosuppressive pathways (3). In humans, monocytes are conventionally classified into three principal subsets based on surface expression of CD14 and CD16: classical (CD14^+^ CD16^-^), intermediate (CD14^+^ CD16^+^), and non-classical (CD14^-^ CD16^+^) monocytes (4). Parallel subsets in murine systems include classical (Ly6C^++^ CD43^+^), intermediate (Ly6C^++^ CD43^++^), and non-classical (Ly6C^+^ CD43^++^) phenotypes. The expression of chemokine receptors such as CCR2 and CX3CR1 provides additional resolution of monocyte identity: CCR2 is predominantly associated with CD14^+^ monocytes, whereas CX3CR1 is highly expressed on the CD16^+^ subset (5, 6).

Beyond their role as precursor cells, monocytes actively shape immune landscapes through the secretion of pro-inflammatory and regulatory cytokines and chemokines. Perturbations in monocyte frequency, phenotype, or function have been implicated in immune dysregulation and the pathogenesis of diverse pathological states, including cancer, autoimmunity, and chronic inflammation (7, 8). As such, elucidating the immunoregulatory mechanisms of monocytes—particularly in the tumor microenvironment—may uncover novel therapeutic avenues and diagnostic biomarkers. This review summarizes current understanding of monocyte heterogeneity, effector functions, and translational relevance, providing conceptual and clinical insights into their roles in tumor immunology and anti-tumor therapy.

## 2 Overview of monocytes

Classical monocytes represent the predominant subset of circulating mononuclear phagocytes and exhibit a high degree of plasticity in both homeostatic and inflammatory contexts. Upon entering peripheral tissues, these cells can function as antigen transporters to draining lymph nodes or differentiate into macrophages that supplement resident populations (9, 10). Without the need for tissue migration, classical CD14^+^CD16^-^ monocytes may mature into intermediate CD14^+^CD16^+^ monocytes and subsequently give rise to non-classical CD14^-^CD16^+^ monocytes (7, 11). Non-classical monocytes possess distinct patrolling behavior, migrating along the vascular endothelium to surveil for and clear cellular debris, thus contributing to vascular homeostasis (5, 12). Across subsets, monocytes express pattern recognition receptors (PRRs), including Toll-like receptors (TLRs), that facilitate the detection of pathogens. Upon activation, monocytes initiate robust immune responses through cytokine secretion, antigen presentation, and orchestration of downstream inflammatory cascades. Importantly, they also exhibit anti-inflammatory capabilities via the production of immunosuppressive cytokines and lipid mediators, underscoring their dual regulatory role in immune modulation and tissue repair (13).

Each monocyte subset harbors unique functional attributes. Classical monocytes are potent producers of proinflammatory mediators such as IL-6, IL-8, CCL2, and CCL3, thereby facilitating immune cell recruitment to sites of infection or injury (14, 15). Intermediate monocytes exhibit heightened responsiveness to lipopolysaccharide (LPS) stimulation, resulting in robust secretion of IL-1β and TNF-α (14). In contrast, non-classical monocytes—marked by high CD16 expression—are adept at mediating antibody-dependent phagocytosis and play a critical role in immune surveillance (16, 17). Altogether, monocytes undergo a dynamic developmental trajectory originating from the bone marrow, characterized by phenotypic transitions, vascular surveillance, and tissue-specific functional specialization. Their differentiation into macrophages often yields cells with features reminiscent of tissue-resident macrophages, yet they retain monocyte-derived signatures and exhibit context-dependent responsiveness in inflammatory milieus (18, 19). A comprehensive understanding of monocyte heterogeneity and function is thus pivotal for elucidating tumor immunology and the immunopathogenesis of various diseases (**Supplementary Table 1**).

Accumulating fate-mapping and single-cell transcriptomic studies demonstrate that many organs harbor long-lived embryonically derived tissue-resident macrophages (TRMs) that self-renew independently of circulating monocytes, whereas inflammatory cues recruit bone-marrow–derived monocytes that differentiate into macrophages *de novo* (20, 21). TRMs (Kupffer cells in the liver, microglia in the brain, and alveolar macrophages in the lung) possess developmentally imprinted chromatin landscapes and oxidative-phosphorylation-biased metabolisms that endow them with niche-specific homeostatic functions (20, 22). By contrast, monocyte-derived macrophages rely on CCR2-mediated egress, exhibit glycolytic wiring, and are highly plastic within the tumor microenvironment (TME) (23, 24). Recent work reveals that monocyte-derived TAMs preferentially up-regulate immunosuppressive molecules such as PD-L1 and VEGF-A, whereas TRMs often contribute to antigen presentation and phagocytosis (25, 26); consequently, therapeutic strategies diverge. CCR2 antagonists (cenicriviroc) or anti-CCL2 antibodies effectively curb monocyte-derived TAM accumulation, whereas CSF1R inhibitors or PI3K-γ blockade are required to reprogram CSF1-dependent TRMs (27, 28). Recognizing these ontogenic distinctions is therefore critical for designing combination immunotherapies that simultaneously deplete suppressive monocyte-derived TAMs and re-educate pro-tumor TRMs.

## 3 Roles of monocytes in tumor immune regulation

Monocytes are highly plastic components of the innate immune system and play pivotal, context-dependent roles in tumor immunity. Rather than being functionally static, monocytes undergo profound reprogramming in response to cues from TME, which intricately “educates” them toward either pro-inflammatory or immunosuppressive phenotypes. This dynamic plasticity renders monocytes a double-edged sword: while they may be activated to initiate antitumor immunity, they can also be co-opted to facilitate tumor progression and immune evasion (29, 30). Deciphering the molecular and cellular mechanisms underlying this phenotypic shift is essential for the rational design of next-generation immunotherapies. Upon recruitment into the TME, monocytes can differentiate into several immunologically distinct cell subsets, including TAMs, dendritic cells (DCs), and myeloid-derived suppressor cells (MDSCs) (17, 31). TAMs, as phagocytic effector cells, contribute to pathogen clearance and tissue remodeling but are frequently polarized into tumor-promoting M2-like states within tumors (32). DCs function as professional antigen-presenting cells that initiate and amplify adaptive immune responses, particularly T cell priming (33). In contrast, MDSCs represent a heterogeneous population of immature myeloid cells that potently suppress T cell activation and proliferation, thereby impairing antitumor immune surveillance and fostering immune tolerance (34). These monocyte-derived lineages orchestrate a complex immunological network that critically shapes tumor evolution and therapeutic response.

### 3.1 Anti-tumor functions of monocytes

#### 3.1.1 Reactive oxygen species and nitric oxide–mediated cytotoxicity

Activated monocytes exert direct cytotoxic effects on tumor cells through the metabolic reprogramming of the L-arginine pathway, leading to the production of reactive nitrogen intermediates (35). These intermediates, particularly nitric oxide (NO), emerged as key effector molecules and signaling mediators, underscoring NO’s central role in macrophage-mediated tumor suppression. This mechanistic insight provided a foundation for recognizing NO not only as a cytotoxic agent but also as a pivotal molecular messenger within antitumor immunity. Besides, monocytes differentiate into classically activated M1 macrophages to mount a potent antitumor response. M1 macrophages secrete a repertoire of pro-inflammatory cytokines, including IL-12, TNF-α, and (IL-1β, which collectively orchestrate robust immune activation (36). These cytokines enhance the cytolytic capacity of CD8^+^ T cells by promoting their activation and clonal expansion, thereby amplifying the adaptive immune response against tumor cells.

#### 3.1.2 Antibody-dependent cellular cytotoxicity

**Figure 1:**
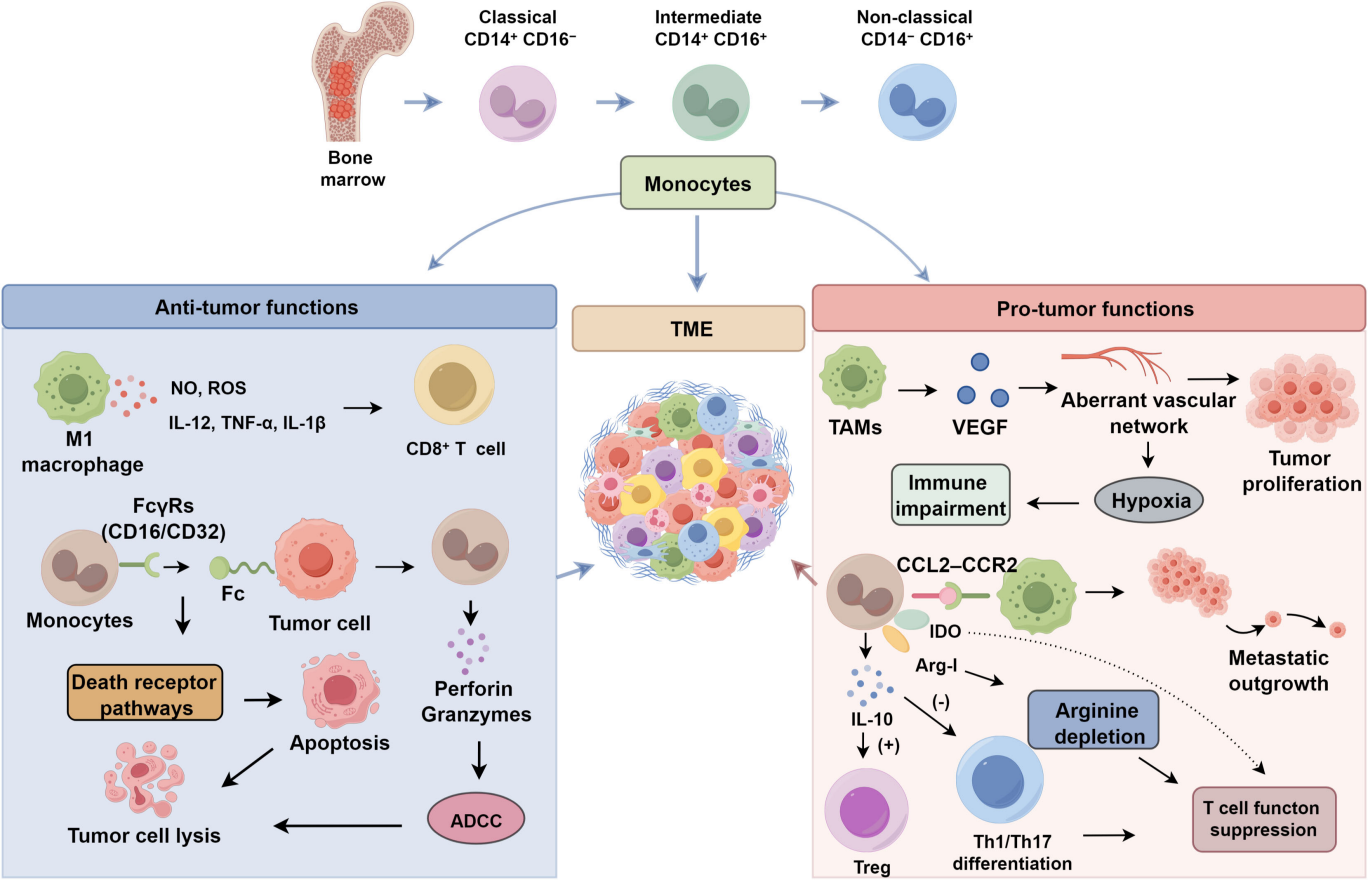
Dual roles of monocytes in tumor immunity.

Monocytes are capable of recognizing the Fc region of therapeutic monoclonal antibodies bound to tumor cell surfaces via surface expression of Fc gamma receptors (FcγRs) such as CD16 and CD32 (37). This interaction subsequently triggers the release of cytotoxic mediators, such as perforin and granzymes, or induces apoptosis through death receptor pathways, culminating in direct tumor cell lysis. The indispensable role of FcγRs in mediating *in vivo* anti-tumor efficacy of therapeutic antibodies was further substantiated by utilizing FcγR-knockout mouse models to establish definitive causal links (38). These findings provided robust experimental support for the pivotal contribution of monocyte/macrophage-mediated ADCC in antibody-based cancer therapy. Although the central work lies in the immunosuppressive microenvironment, their co-culture assays involving monocytes and tumor cells offer compelling evidence of monocyte-mediated direct cytostatic and cytotoxic effects on specific tumor cell lines—an approach widely adopted for *in vitro* validation of ADCC (38–40). Overall, monocytes and macrophages form a multilayered immunological barrier against tumors. Beyond their innate cytotoxic capacity driven by metabolic intermediates, these cells serve as key orchestrators of the tumor microenvironment by secreting cytokines that regulate immune dynamics and initiate adaptive responses (41). The integration of these diverse functional modalities positions monocytes/macrophages as central hubs in the coordination of anti-tumor immunity (**Figure 1**).

### 3.2 Pro-tumorigenic roles of monocytes

Monocytes contribute to immune evasion primarily through their recruitment to the TME, where they differentiate into TAMs and exert potent immunosuppressive effects (29, 42). Monocyte-derived TAMs secrete pro-angiogenic factors such as VEGF, facilitating the formation of an aberrant vascular network (43). This dysregulated vasculature not only supports tumor proliferation but also fosters a hypoxic and acidic milieu that impairs effector immune cell function (44). Furthermore, monocytes and macrophages establish a self-sustaining loop via the CCL2–CCR2 axis, maintaining their presence within metastatic sites and promoting metastatic outgrowth (45, 46). Notably, monocytes directly modulate T cell responses through both cytokine secretion and cell–cell contact. IL-10, abundantly produced by monocytes, inhibits the differentiation of Th1 and Th17 cells while promoting Treg development, thereby attenuating antitumor immunity (17). DeNardo et al. (47) highlighted the dualistic role of monocyte-derived macrophages in cancer immunity, emphasizing IL-10 as a central mediator of immunosuppression and resistance to both chemotherapy and immune checkpoint blockade. Elevated expression of Arg-I in monocytes/macrophages, leading to arginine depletion within the TME. As arginine is critical for T cell activation and proliferation, its scarcity results in T cell cycle arrest and functional impairment (48). In parallel, myeloid cells express indoleamine 2, 3-dioxygenase (IDO), which catalyzes the degradation of tryptophan into immunosuppressive kynurenine metabolites that directly suppress T cell activity (49). These findings establish monocytes as key immunoregulatory players: not only as suppressors of T cell function (IL-10), but also as metabolic modulators (Arg-I and IDO), and facilitators of tumor progression (VEGF-driven angiogenesis). Such mechanistic insights provide a strong rationale for developing monocyte/TAM-targeted therapeutic strategies, including inhibitors of CCR2 and CSF-1R, to disrupt these tumor-promoting pathways.

## 4 Applications of monocytes in tumor immunotherapy

Alterations in the frequency and functional phenotype of circulating monocytes have been shown to significantly influence cancer progression and clinical outcomes in patients with malignancies (17). As a result, monocytes have emerged as promising therapeutic targets in the context of cancer immunotherapy. Notably, monocytic myeloid-derived suppressor cells (M-MDSCs), which originate from monocyte precursors, exhibit potent immunosuppressive properties and actively facilitate tumor growth by dampening antitumor immune responses (50, 51). Therapeutic strategies aimed at depleting or functionally reprogramming MDSCs have demonstrated potential in enhancing the efficacy of immune-based treatments. The elevated levels of CCR2^+^ inflammatory monocytes in the peripheral blood of patients with pancreatic cancer correlated with poorer survival outcomes, providing direct clinical rationale for targeting this axis (52). Thus, the therapeutic efficacy of CCR2- or CSF1R-directed approaches has been inconsistent across studies, and their clinical benefit has often been limited by the adaptive resilience of the tumor myeloid niche. Emerging evidence suggests that selective blockade of monocyte/macrophage recruitment or survival may induce compensatory infiltration of other suppressive myeloid populations, including neutrophils and non-targeted MDSC subsets, which can sustain immunosuppression and tumor progression (53).

Researchers have validated the therapeutic potential of innate immune cell–based adoptive therapies: autologous monocytes activated ex vivo with IFN-α and IFN-γ were safely infused into patients, displaying sustained persistence and inducing both systemic and intratumoral immune responses (54). Tumor-associated macrophages have been shown to potentiate responses to ICIs in preclinical models of colorectal adenocarcinoma, breast cancer, melanoma, and osteosarcoma. Mechanistically, blockade of the PD-1/PD-L1 axis can reprogram TAMs toward a pro-inflammatory phenotype, thereby enhancing effector T cell activation and improving tumor control (55, 56). Drug-loaded nanocarriers selectively taken up by monocytes/macrophages could efficiently deliver therapeutic agents to the tumor site, thereby overcoming the limitations of conventional chemotherapy (57). Moreover, the presence of inflammatory macrophages has been associated with favorable responses to ICIs in patients with urothelial carcinoma and non-small cell lung cancer (NSCLC) (58). Nonetheless, clinical monitoring of macrophage subsets remains technically challenging, primarily due to limitations in specific markers. CD68, a widely used pan-macrophage marker, lacks specificity—it is also expressed by granulocytes, dendritic cells, fibroblasts, endothelial cells, and certain lymphocyte subsets—and does not distinguish between macrophage subpopulations (59).

### 4.1 TCM compounds reprogram monocytes toward antitumor immunity

Bioactive compounds derived from TCM exert potent immunomodulatory effects on monocytes, thereby enhancing antitumor immunity across multiple cancer types, including colorectal cancer, hepatocellular carcinoma (HCC), breast cancer, and lung cancer. Mechanistically, these compounds primarily function by reprogramming monocyte differentiation and polarization, shifting them from immunosuppressive phenotypes toward pro-inflammatory, tumoricidal states (60). This process involves the coordinated regulation of key inflammatory signaling pathways, including NF-κB, STAT, PI3K/AKT, MAPK, and inflammasome-associated cascades.

Curcumin, a polyphenolic compound extracted from *Curcuma longa*, represents one of the most extensively studied TCM monomers in tumor immunology. In monocytes, curcumin exerts dual regulatory effects depending on the tumor context. It suppresses aberrant NF-κB activation by inhibiting IκB kinase (IKK) phosphorylation, thereby reducing the production of immunosuppressive cytokines such as IL-10 and TGF-β within the tumor microenvironment (61). Concurrently, curcumin enhances the expression of pro-inflammatory mediators, including TNF-α, IL-12, and inducible nitric oxide synthase (iNOS), through activation of the p38 MAPK and JNK pathways. This reprogramming promotes differentiation toward M1-like macrophages, leading to increased nitric oxide and ROS production, which directly mediate tumor cell cytotoxicity (62, 63). In models of colorectal and breast cancer, curcumin-treated monocytes also exhibit enhanced antigen-presenting capacity via upregulation of MHC class II and co-stimulatory molecules (CD80/CD86), thereby amplifying CD8^+^ T cell–mediated responses (64, 65).

Flavonoid compounds, such as quercetin, further contribute to monocyte-mediated antitumor effects through modulation of the PI3K/AKT and STAT3 signaling axes. In tumor-associated monocytes, persistent activation of STAT3 is a key driver of immunosuppression and M2 polarization (66, 67). Quercetin inhibits STAT3 phosphorylation and nuclear translocation, thereby attenuating IL-6–dependent signaling and reducing the expression of Arg-1 and IDO, two critical enzymes involved in metabolic immune suppression (68). Simultaneously, quercetin activates AMPK signaling, leading to metabolic reprogramming of monocytes toward glycolysis-dependent pro-inflammatory states, which are associated with enhanced tumoricidal activity. In lung cancer and melanoma models, this metabolic shift correlates with increased secretion of IL-1β and IL-12 and reduced recruitment of Tregs, ultimately reshaping the tumor immune microenvironment (69, 70).

Saponins, particularly ginsenosides derived from *Panax ginseng* (Rg3 and Rh2), have also demonstrated significant capacity to modulate monocyte function. Ginsenoside Rg3 has been shown to inhibit the CCL2–CCR2 chemokine axis, thereby reducing the recruitment of inflammatory monocytes into tumor sites and limiting their subsequent differentiation into tumor-associated macrophages (71, 72). At the same time, ginsenosides promote the activation of Toll-like receptor (TLR) signaling pathways, particularly TLR4/MyD88-dependent cascades, leading to enhanced NF-κB activation in a context-dependent manner that favors antitumor inflammation (73). This activation induces the production of IL-12 and IFN-γ while suppressing IL-10, thereby restoring the balance between pro- and anti-inflammatory signaling. Moreover, ginsenosides have been reported to regulate the NLRP3 inflammasome in monocytes, facilitating caspase-1 activation and IL-1β maturation, which further amplifies innate immune responses against tumor cells (74, 75).

Importantly, several TCM-derived compounds also influence monocyte–tumor crosstalk by targeting immune checkpoint–related pathways. For instance, curcumin and ginsenosides have been shown to downregulate PD-L1 expression on monocytes and macrophages through inhibition of the PI3K/AKT/mTOR and STAT3 pathways, thereby enhancing T cell activation and overcoming immune escape. Additionally, these compounds can modulate the tumor metabolic microenvironment by inhibiting IDO-mediated tryptophan metabolism and lactate-induced immunosuppression, further reinforcing monocyte-driven antitumor immunity (76). Overall, TCM-derived bioactive molecules represent a promising class of immunomodulators capable of reprogramming monocytes through multi-target and multi-pathway mechanisms. By integrating inflammatory signaling modulation, metabolic reprogramming, and immune checkpoint regulation, these compounds provide a mechanistic basis for the development of novel combinatorial strategies in cancer immunotherapy.

## 5 Conclusion

Monocytes are highly plastic innate immune cells that exert dual and context-dependent roles in tumor immunity. They can support antitumor defense through nitric oxide and reactive oxygen species production, Fc receptor-mediated cytotoxicity, antigen presentation, and the secretion of inflammatory cytokines such as IL-12 and TNF-α. However, under tumor-derived cues, monocytes may differentiate into immunosuppressive populations, including tumor-associated macrophages and monocytic myeloid-derived suppressor cells, thereby promoting angiogenesis, T-cell dysfunction, and immune escape. Mechanistically, these protumor effects are driven by pathways such as CCL2–CCR2-dependent recruitment, VEGF-mediated vascular remodeling, IL-10- and TGF-β-mediated immune suppression, and metabolic restriction of T cells through arginase-1 and indoleamine 2, 3-dioxygenase.

From a therapeutic perspective, future studies should focus on defining the ontogeny and functional specialization of monocyte/macrophage subsets and identifying the molecular circuits governing their reprogramming in tumors. Particular attention should be given to macrophage-intrinsic PD-1/PD-L1 signaling, STAT3- and NF-κB-dependent transcriptional programs, and metabolic pathways involving arginine, tryptophan, and lipid metabolism, which collectively shape immunosuppressive phenotypes. Translationally, selective blockade of monocyte recruitment axes such as CCL2–CCR2 or CSF1–CSF1R, re-education of macrophages toward inflammatory states, and nanoparticle-based drug delivery may improve therapeutic precision. In addition, rational combination strategies with immune checkpoint blockade may be required to overcome myeloid-mediated resistance and enhance durable antitumor immunity.
